# Alternative positioning method for the superior–inferior axial shoulder projection – the Lewis modification

**DOI:** 10.1002/jmrs.583

**Published:** 2022-04-15

**Authors:** Kim Lewis

**Affiliations:** ^1^ Radiology Taranaki District Health Board New Plymouth New Zealand

**Keywords:** ​glenoid cavity imaging, lewis modification, superior–inferior axial shoulder x–ray

## Abstract

The superior–inferior (SI) axial shoulder view is an important part of shoulder imaging. It provides a true orthogonal view to the anterior–posterior (AP) shoulder projection and is a supplementary view to the lateral scapula view. When positioned correctly, the glenohumeral joint is visualised with the superior and inferior aspects of the glenoid superimposed to demonstrate the true relationship between the glenoid and humerus. Positioning for the SI axial view is challenging. Often the glenoid is not superimposed on resulting images, and the glenohumeral relationship cannot be assessed accurately. Some positioning texts do not demonstrate the SI axial view, opting instead for the inferior–superior (IS) view. When the SI axial view is included, bony landmarks are not provided to assist medical imaging technologists (MITs) with accurate positioning. This paper outlines a proposed modification using bony landmarks that can assist MITs in positioning their patients for this important view and obtain diagnostic images that demonstrate the glenoid in true profile.

## Graphical abstract

The superior‐inferior (SI) axial shoulder view is an important part of shoulder imaging. When positioned correctly the gleno‐humeral joint is visualised with the superior and inferior aspects of the glenoid superimposed to demonstrate the true relationship between the glenoid and humerus. This paper outlines a proposed modification using bony landmarks that can assist MITs in positioning their patients for this important view and obtain diagnostic images that demonstrate the glenoid in true profile.

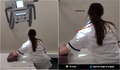

## Introduction

Two bones comprise the shoulder girdle, the scapula and the clavicle. The proximal aspect of the humerus is included as part of the shoulder joint as it articulates with the scapula but is considered part of the upper limb.^1^ The glenoid cavity is on the lateral aspect of the scapula and articulates directly with the humerus. It has an irregular shape and sits at an angle of approximately 5–15°.^2^ The purpose of the superior–inferior (SI) axial shoulder view is to demonstrate the relationship between the humerus and the glenoid cavity and provide additional information when assessing for glenohumeral instability.^1^, ^2^, ^3^, ^4^, ^5^ It is a supplementary view alongside the lateral scapula for obtaining orthogonal images to the anterior–posterior shoulder view.^3^

**Figure 1:**
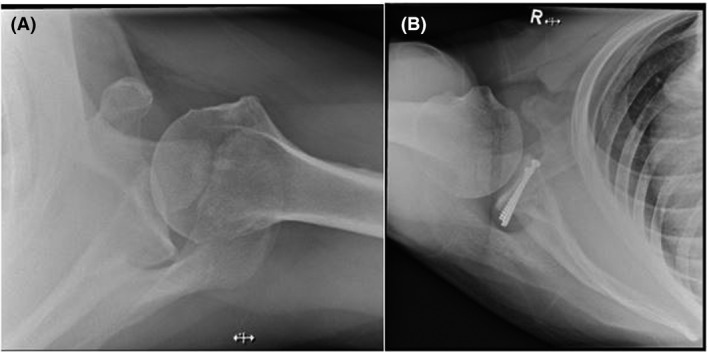
(A) It demonstrates a typical superior–inferior axial shoulder projection where the superior and inferior margins of the glenoid are not superimposed, and the glenohumeral joint is not open. (B) The superimposed superior margins demonstrate the joint open.

When the SI axial view is correctly obtained, the glenohumeral joint is open with the inferior and superior aspects of the glenoid superimposed on itself. This allows the visualisation of the relationship between the humerus and the glenoid (Fig. 1).^1^, ^2^, ^4^ A database search of Pubmed and EBSCO for the phrase ‘axial shoulder’ found one study related to shoulder imaging on x‐ray. This study found most conventional axial radiographs do not adequately demonstrate superimposition of the glenoid.^2^

Positioning for the SI axial shoulder view can be challenging. Some positioning texts omit this common position, opting to demonstrate the inferior–superior (IS) axial projection instead.^5^, ^6^ Three sources were found that included the SI axial view.^1^, ^3^, ^7^ In these sources, the patient is positioned by extending the affected shoulder over the detector and a straight ray or a tube angle towards the elbow of 5–15° is used. The resultant images demonstrated in these sources do not visualise the glenoid in superimposition so therefore do not fully demonstrate the glenohumeral joint relationship, despite stating this as a positioning requirement.^1^, ^3^, ^4^, ^6^ Additionally, these sources do not provide guidelines on how to correct the image if the glenoid is not superimposed.^1^, ^3^, ^6^

**Figure 2:**
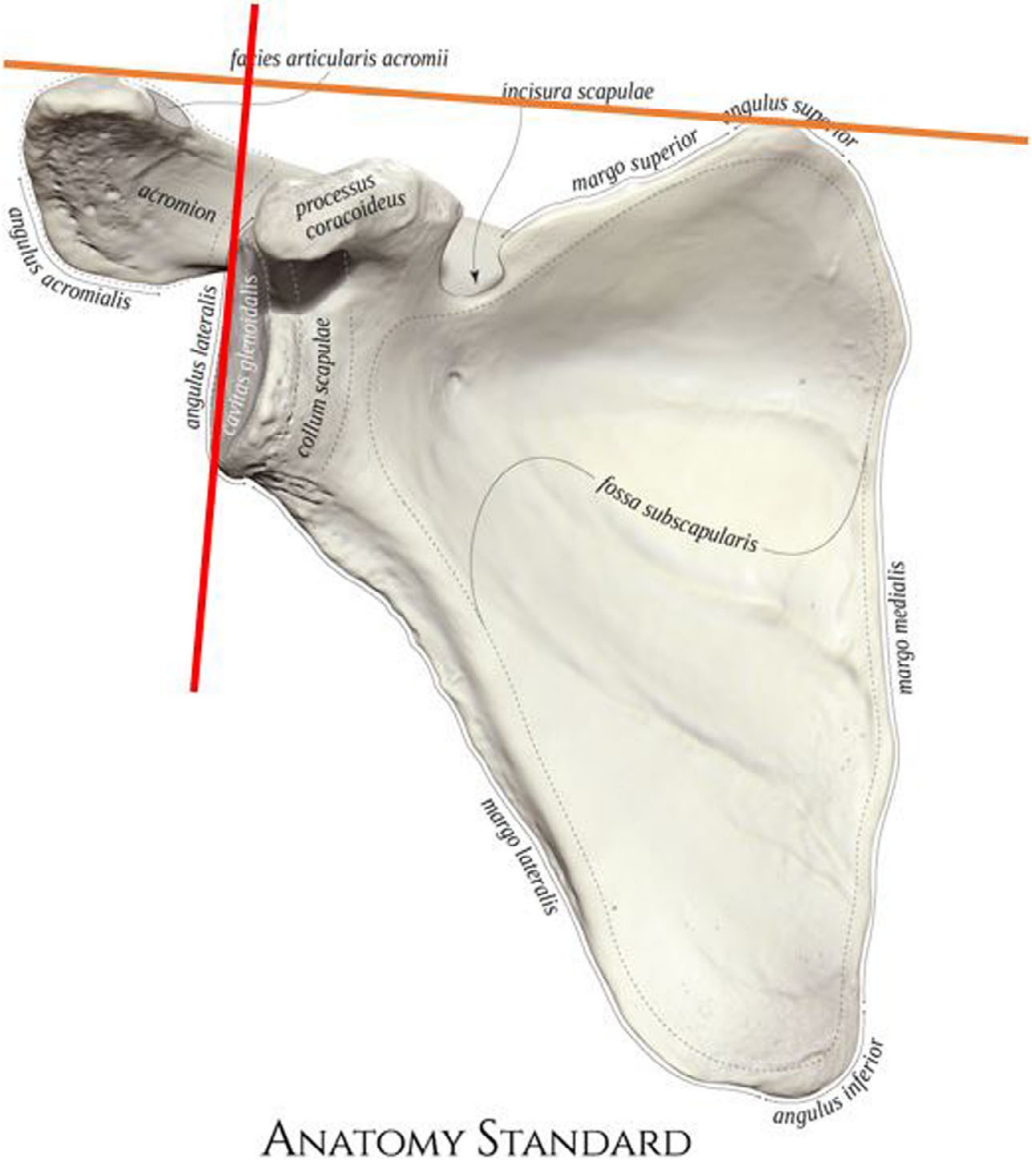
Scapula anatomy showing the imaginary AC joint to superior angle line and the perpendicular glenoid line.^8^ Images adapted under Creative Commons licence CC BY‐NZ 4.0. [Colour figure can be viewed at wileyonlinelibrary.com]

McQuillen Martensen (2020) do provide information on correcting axial shoulder images; however, these instructions are for the IS axial shoulder view and can be challenging to interpret for the SI view.^4^ The Lewis modification provides bony landmarks to more easily assess the angle needed to assist in superimposing the glenoid and achieving appropriate images. Corrective actions specific to the SI axial view are given to assist with repeat imaging. Note: All images in this manuscript, except Figure 2, were obtained by the author.

## Proposed Method

While researching methods of positioning for the SI axial view, it was noted that the angle of the glenoid was nearly perpendicular to an imaginary line drawn that intersects the AC joint and the superior angle of the scapula (Fig. 2). The proposed Lewis modification makes use of this imaginary line to assist with positioning patients.

### Positioning for the Lewis modification

The patient sits at the end of the X‐ray table, with the table height at approximately the level of the patients' hip to allow the patient to lean towards the affected side. The image receptor (IR) is flat on the table with one edge against the patient's waist. The patient flexes their elbow to 90° and abducts their affected arm to their full range of motion. The patient leans laterally over the IR, placing their elbow as lateral as they can. It is important that the patient leans as far they can to aid with ensuring their glenohumeral joint is projected on the IR. Prop the affected arm on a sponge to rotate the glenoid cavity more vertical and away from the chest wall. Ensure the patients spine is kept straight and they do not lean forwards or backwards. Turn the patients head away from the affected side.

**Figure 3:**
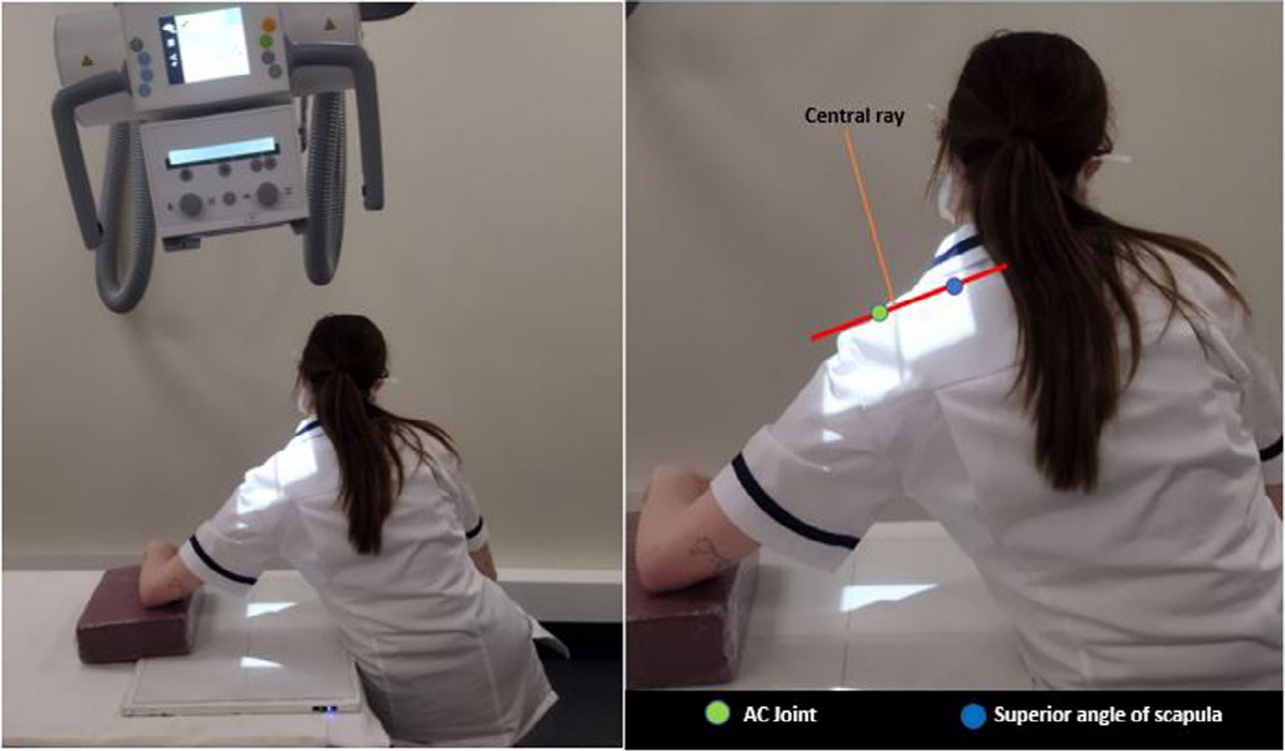
Example of the optimal positioning for the Lewis modification. [Colour figure can be viewed at wileyonlinelibrary.com]

Feel for the AC joint and the superior angle of the scapula and imagine a line intersecting these landmarks. Angle the tube to be perpendicular to this line. Centre the central ray to the AC joint as this will intersect through the glenohumeral joint (Fig. 3).

This position will create an increased object to image receptor distance (OID). To reduce the OID, once the patient is positioned, raise the height of the table while ensuring the anatomy remains projected on the detector. This may change the angle of the tube slightly. It is recommended the tube height is increased to 120–150 cm to minimise magnification. The patients range of motion and resulting tube angle may cause the glenoid to overlay part of the chest wall, A kV of 65–70 is recommended to ensure adequate penetration. Because this modification creates an airgap, it is not necessary to use a grid. Collimate to include the proximal 1/3 of the humerus, the glenohumeral joint, coracoid and lateral aspects of the scapula.

**Figure 4:**
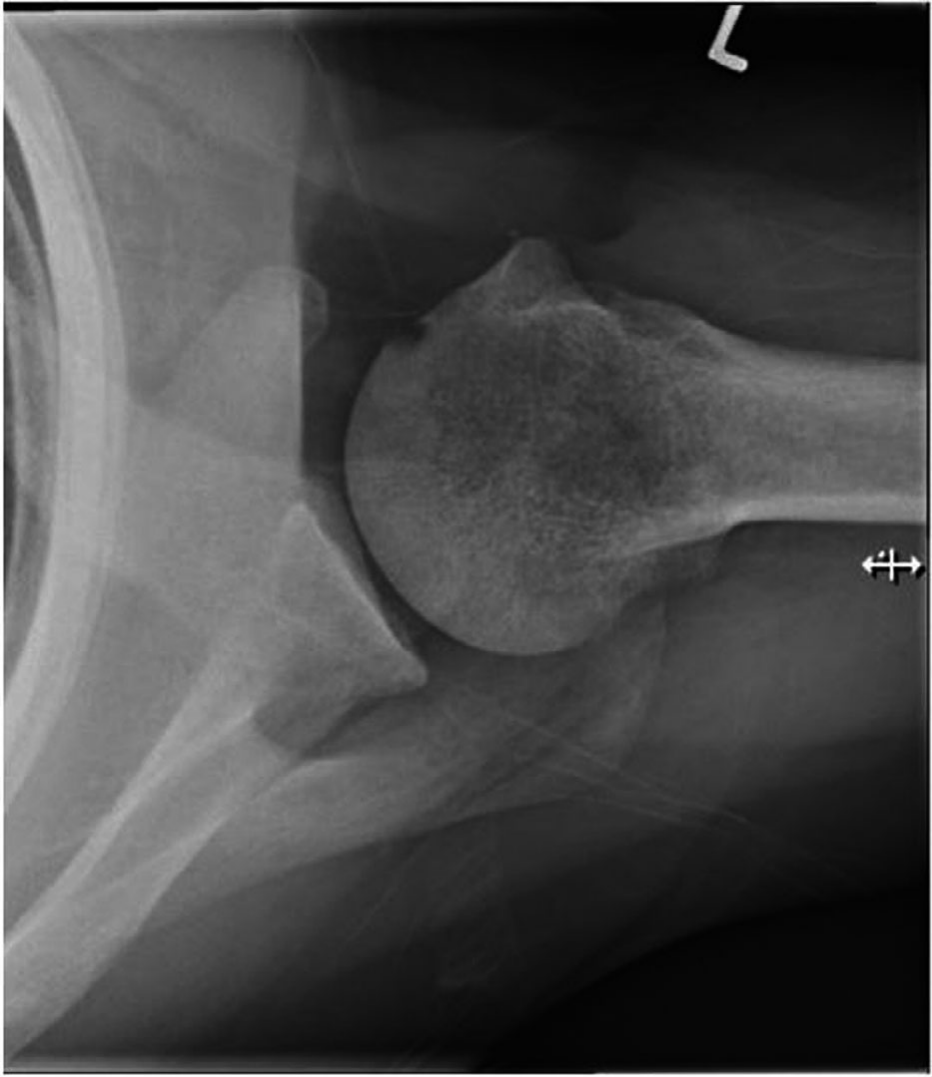
Correctly positioned superior–inferior axial shoulder using the Lewis modification.

The resulting image demonstrates the superior and inferior aspects of the glenoid superimposed on itself (Fig. 4).

### Position correction

**Figure 5:**
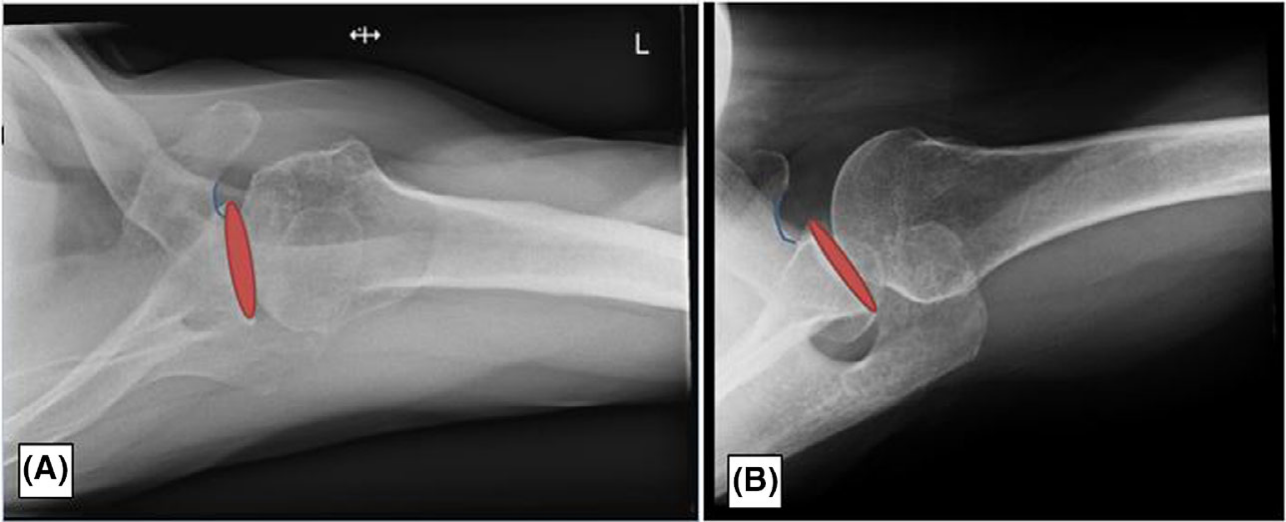
Images demonstrating the relationship of the base of the coracoid to the glenoid cavity to assist with correcting the central ray. The base of coracoid is intersecting or lateral to the glenoid cavity (A) or the base of coracoid is medial to the glenoid cavity (B). [Colour figure can be viewed at wileyonlinelibrary.com]

If the glenoid cavity is not superimposed, correction to the positioning can be made by identifying the base of the coracoid and correcting the central ray based on its position in relation to the glenoid. If the base of the coracoid is intersecting or lateral to any aspect of the glenoid cavity, the angle of the central ray should be decreased. If the base of the coracoid is medial to the glenoid cavity, the angle of the central ray should be increased (Fig. 5).

## Conclusion

Many SI axial shoulder images do not demonstrate the glenohumeral joint accurately, with many images lacking superimposition of the glenoid. SI axials views are challenging to position and existing texts do not provide external landmarks to assist with ensuring MITs can position accurately on the initial image. The proposed Lewis modification is a method of positioning for the SI axial shoulder view that utilises landmarks to assist with accurate positioning to ensure superimposition of the glenoid cavity. This aids with minimising repeats and ensures that SI axial shoulder views demonstrate anatomy accurately. However, further study is needed to evaluate the effectiveness of this proposed modification.

## Funding information

No funding was sought to complete this paper.

## Conflict of Interest

There are no conflicts of interest to disclose.

## Ethical Approval

Ethics approval was not sought as this was an adaptation on an existing protocol.
